# Mechanistic Study of Matrix Stiffness Promoting Lymph Node Metastasis in Cervical Cancer by Regulating NETs Formation via Piezo1

**DOI:** 10.3390/ijms27125431

**Published:** 2026-06-16

**Authors:** Lanyue Zhang, Zhuqing Ouyang, Jiarong Tan, Wei Li, Fujin Shen

**Affiliations:** Department of Obstetrics and Gynecology, Renmin Hospital of Wuhan University, 239 Jiefang Road, Wuchang District, Wuhan 430060, China; 2023283020171@whu.edu.cn (L.Z.); 18779270196@163.com (Z.O.); tjr072202@163.com (J.T.); weili079@163.com (W.L.)

**Keywords:** matrix stiffness, cervical cancer, neutrophil extracellular traps, piezo1, lymphatic metastasis

## Abstract

Cervical cancer is a common gynecological malignancy, with a 5-year survival rate of only 17% for recurrent or metastatic cases. Increased extracellular matrix stiffness, a key change in the tumor mechanical microenvironment, promotes tumor metastasis via mechanotransduction. Piezo1, a mechanosensitive cation channel, senses matrix stiffness and converts mechanical signals into intracellular chemical signals. Neutrophil extracellular traps (NETs) are overformed in tumors, but the mechanism by which matrix stiffness regulates NETs in cervical cancer remains unclear. We detected matrix stiffness and related protein expression in cervical cancer tissues using atomic force microscopy and histochemical staining. Polyacrylamide gel models were used to culture HeLa/SiHa cells, with transcriptome sequencing and ELISA to analyze IL-8 expression. NETs were induced from human peripheral blood neutrophils, and their effect on lymphatic endothelial cells was evaluated. A TC-1 mouse model was used to verify in vivo effects, and Western blot/ELISA explored the *Piezo1/NF-κB* pathway. Higher Young’s modulus, increased *α-SMA/Collagen I* expression and collagen content in metastatic cervical cancer tissues. High matrix stiffness activated *Piezo1/NF-κB*, upregulated *IL-8*, induced NETs, and enhanced lymphatic endothelial cell tube formation/migration. BAPN reduced tumor stiffness, inhibited metastasis, and decreased NETs in mice. Knocking down Piezo1 blocked *NF-κB* activation and *IL-8* upregulation. High matrix stiffness activates *Piezo1/NF-κB* to promote *IL-8* secretion and NETs formation, enhancing lymphangiogenesis and cervical cancer metastasis, providing a new target for advanced cervical cancer treatment.

## 1. Introduction

Cervical cancer is one of the most common malignant tumors of the female reproductive system. Due to the unique pelvic anatomical structure, the dissemination of cervical cancer cells primarily relies on the lymphatic system, with a predilection for invading pelvic and para-aortic lymph nodes. Accumulating clinical evidence indicates that cervical cancer patients with lymph node metastasis have a significantly increased risk of postoperative recurrence, with a sharp 40% decrease in the 5-year overall survival rate [1]. Based on this, the 2018 revised International Federation of Gynecology and Obstetrics (FIGO) staging system has incorporated lymph node involvement into the staging criteria for stage IIIC and above, further highlighting the critical value of lymph node status in cervical cancer staging and prognosis assessment [2]. Therefore, in-depth elucidation of the molecular regulatory mechanisms underlying cervical cancer lymph node metastasis and identification of potential intervention targets have become important scientific propositions for improving clinical outcomes in advanced patients.

In recent years, the remodeling of physical properties in the tumor microenvironment has been increasingly recognized as a crucial mechanism driving tumor malignant progression [3]. Among these, increased extracellular matrix stiffness, a typical feature of the mechanical microenvironment, can regulate tumor cell biological behaviors and reshape the tumor immune microenvironment by activating cellular mechanotransduction pathways, thereby providing potential mechanical driving signals for tumor dissemination [4]. Although this phenomenon has been confirmed in various solid tumors, how matrix stiffness participates in regulating cervical cancer metastasis through specific signaling pathways and its specific molecular targets remain to be further elucidated [5]. In this process, the mechanosensitive ion channel protein Piezo1, as a core sensor of extracellular mechanical stimuli, can recognize changes in matrix stiffness and activate downstream signaling cascades such as *NF-κB* and *YAP/TAZ* [6,7,8]. Notably, although *Piezo1* has been confirmed to be involved in regulating cell proliferation, migration, and angiogenesis in malignant tumors such as breast cancer [9] and liver cancer [10], its role in mediating matrix stiffness signals, regulating inflammatory factor networks, and interacting with immune cell functions in cervical cancer remains relatively understudied and requires systematic in-depth investigation.

Among the numerous components of the tumor microenvironment, the abnormal activation of immune regulatory networks has become a key variable determining tumor progression [11]. As reticular fibrous structures released by activated neutrophils into the extracellular space, NETs have attracted significant attention in the field of tumor immunology research in recent years. A large body of evidence indicates that as pro-inflammatory effector molecules, NETs can reshape the tumor microenvironment from multiple dimensions and accelerate tumor malignant progression [12]. Specifically, NETs can systematically promote the distant dissemination of tumor cells through directing their chemotaxis to target organs [13], degrading the extracellular matrix barrier [14], and regulating angiogenesis and lymphangiogenesis. Their central role in promoting tumor metastasis has been gradually established.

Lymphangiogenesis is considered a critical prerequisite for tumor cells to enter the lymphatic circulation and achieve lymph node metastasis [15]. However, whether NETs, as important mediators confirmed to be involved in inflammation-related pro-metastatic processes, affect cervical cancer lymphatic metastasis and the specific mechanisms remain poorly understood. The existing literature suggests that NETs can induce phenotypic remodeling of tumor cells, significantly enhancing their migratory and invasive potential, thereby promoting the occurrence of lymph node metastasis [16]. Our previous studies have confirmed that NETs are significantly upregulated in cervical cancer tissues, and their expression levels are further increased in cervical cancer tissues with lymph node metastasis, suggesting that NETs may act as key bridging molecules and play an important role in the cascade regulation of cervical cancer lymphatic metastasis.

Recent research evidence indicates that increased extracellular matrix stiffness can significantly upregulate the expression level of *IL-8* in tumor cells by activating intracellular mechanical signal transduction pathways [17]. As a classic chemokine, *IL-8* has been confirmed to be one of the key upstream signaling molecules inducing neutrophils to release NETs [18]. Therefore, we propose the following hypothesis: increased matrix stiffness in the tumor microenvironment may upregulate the expression and secretion of *IL-8* by activating the mechanosensitive ion channel protein Piezo1, thereby inducing the formation of NETs and promoting lymph node metastasis of cervical cancer. This study aims to further investigate whether matrix stiffness regulates *IL-8* expression through *Piezo1* to induce NETs formation and promote cervical cancer lymph node metastasis by intervening in the biomechanical signal transduction of cervical cancer matrix stiffness in vitro and in vivo. It will reveal the critical role of the mechanical microenvironment as a “mechanical driver” in the distant metastasis of cervical cancer and provide new therapeutic targets for the treatment of advanced cervical cancer.

## 2. Results

### 2.1. Cervical Cancer Tissues with Lymph Node Metastasis Exhibit Higher Stiffness than Those Without Metastasis

**Figure 1:**
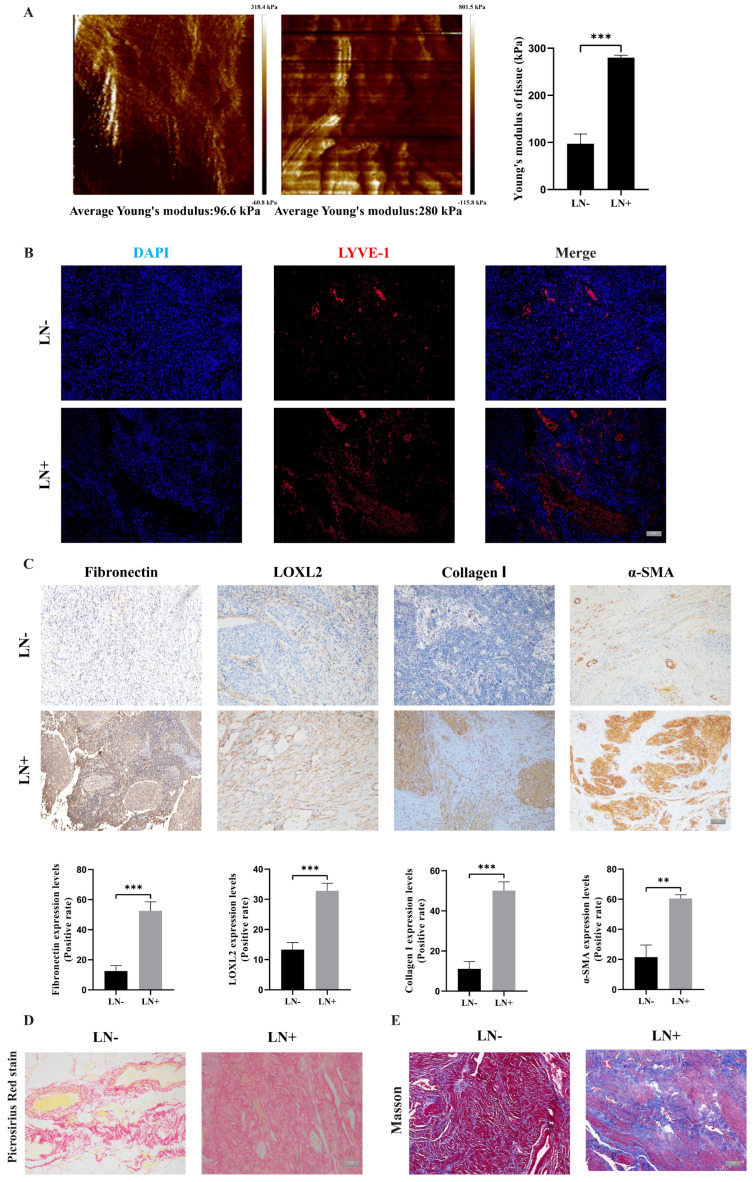
**Cervical cancer tissues with lymph node metastasis exhibit higher stiffness compared to those without lymph node metastasis.** (**A**). Young’s modulus measurement and representative images of cervical cancer tissues with or without lymph node metastasis detected by atomic force microscopy; (**B**). *LYVE-1* immunofluorescence staining of cervical cancer tissues in the two groups (DAPI: blue; *LYVE-1*: red); (**C**). Expression levels of *α-SMA*, *Collagen I*, *LOXL2* and *Fibronectin* in the two groups assessed by immunohistochemical staining and corresponding quantitative analysis; (**D**). Collagen fiber content in the two groups detected by Sirius Red staining; (**E**). Collagen fiber content in the two groups examined by Masson staining (scale bar: 50 μm). Experiments were performed with 20 independent human clinical specimens (*n* = 10 per group, biological replicates), 5 random microscopic fields were captured per sample for quantitative analysis (technical replicates). ** *p* < 0.01; *** *p* < 0.001.

To clarify the effect of matrix stiffness in cervical cancer tissues on lymph node metastasis and its correlation with tissue composition, the Young’s modulus of 20 cervical cancer specimens (10 in the lymph node metastasis group and 10 in the non-metastasis group) was quantitatively measured using atomic force microscopy (AFM). The mean Young’s modulus was 96.6 kPa in the non-metastatic group and 280 kPa in the metastatic group, indicating that cervical cancer tissues with lymph node metastasis possessed significantly higher matrix stiffness (Figure 1A). Given that lymphangiogenesis serves as a critical structural basis for tumor lymph node metastasis, lymphatic vessel density was evaluated in both groups. Immunofluorescence staining for *LYVE-1*, a specific marker of lymphatic endothelium, revealed that lymphatic vessel density was markedly higher in the metastatic group than in the non-metastatic group (Figure 1B). Immunohistochemical staining was further performed to detect the expression of matrix stiffness-related molecules. The expression levels of *α-SMA*, *Collagen I*, *LOXL2*, and *Fibronectin* were significantly elevated in primary cervical cancer lesions of the metastatic group compared with the non-metastatic group (Figure 1C). Sirius Red staining confirmed increased collagen deposition in the stroma of metastatic lesions (Figure 1D), and Masson’s trichrome staining further verified this trend, showing markedly higher stromal collagen content in the metastatic group (Figure 1E). Collectively, increased matrix stiffness in cervical cancer tissues is closely associated with lymph node metastasis.

### 2.2. Increased Matrix Stiffness Upregulates the Expression of Piezo1 and IL-8 in Cervical Cancer Cells and Is Associated with Poor Prognosis

**Figure 2:**
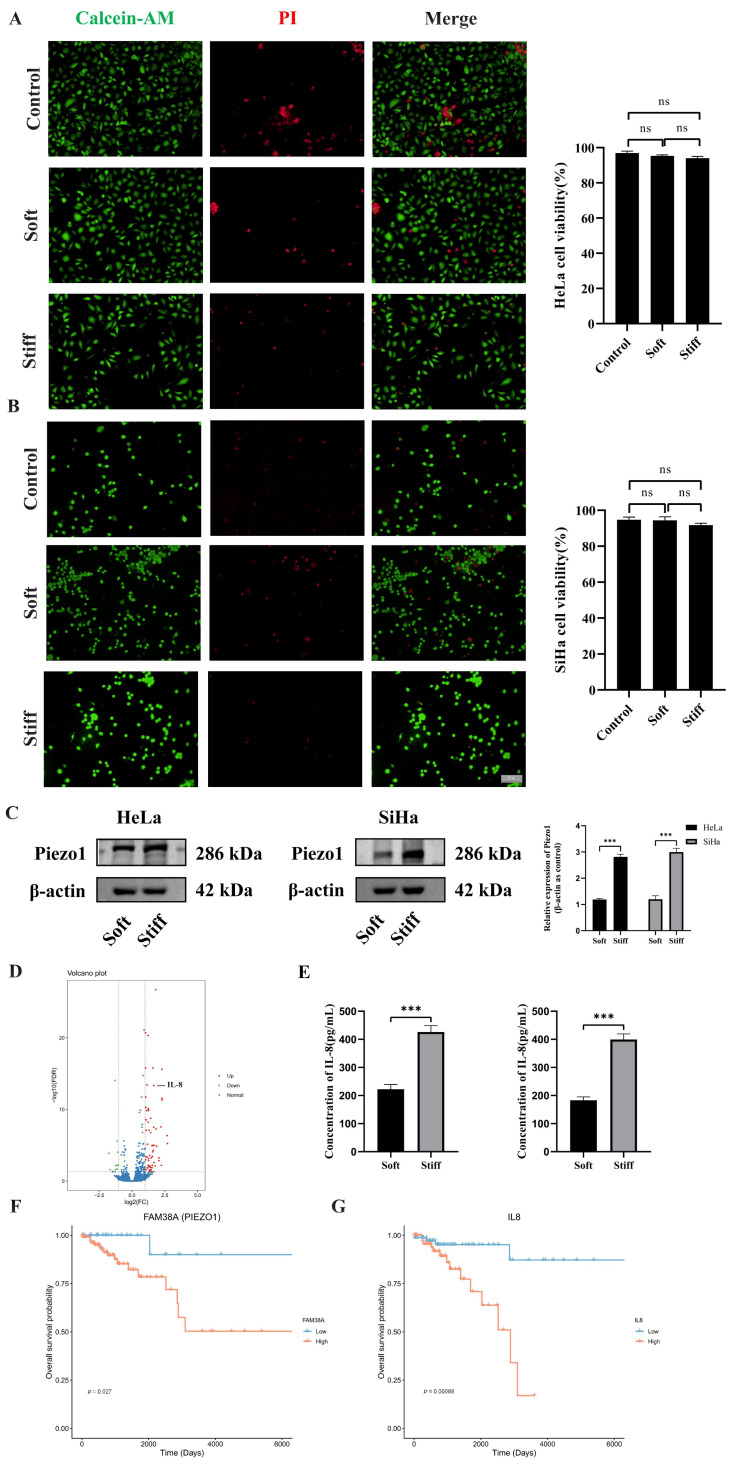
**Matrix stiffness promotes IL-8 and Piezo1 expression in cervical cancer cells without affecting cell viability.** (**A**,**B**). Viability of HeLa and SiHa cells in polyacrylamide hydrogel models detected by live/dead staining, showing representative images and quantitative analysis (scale bar: 50 μm). Green fluorescence indicates live cells, and red fluorescence indicates dead cells; (**C**). Western blot and quantitative analysis of the effect of soft and stiff matrices on Piezo1 expression in HeLa and SiHa cells; (**D**). Transcriptome sequencing technology was used to detect differences in IL-8 RNA expression profiles in cervical cancer cells cultured on soft and stiff matrix gels, with results presented as a volcano plot; (**E**). ELISA assay for *IL-8* secretion levels in the supernatant of cervical cancer cells under different matrix stiffness conditions (Left: HeLa; Right: SiHa); (**F**,**G**). Kaplan–Meier (KM) survival curve comparison of cervical cancer patients with high vs. low expression of *Piezo1* and *IL-8* based on the TCGA-CESC database. All cell-based experiments were performed with 3 independent biological replicates, and ≥3 technical replicates for each test. *** *p* < 0.001, ns: no significance.

An in vitro culture model with polyacrylamide gels of varying matrix stiffness was constructed to mimic the different stiffness microenvironments of cervical cancer cells. HeLa and SiHa cells were seeded onto the gels, and cell viability was assessed by live/dead staining after 3 days of culture to verify whether the model supported cell growth. Results showed that the vast majority of cells were viable (green fluorescence), with very few dead cells (red fluorescence) on both soft and stiff gels, indicating that the model effectively supported normal adhesion and growth of cervical cancer cells (Figure 2A,B). Our previous studies have confirmed that increased matrix stiffness can significantly promote the expression of Piezo1 in cervical cancer cells (Figure 2C). However, whether matrix stiffness further regulates the transcription and protein secretion of downstream genes in tumor cells through mechanotransduction pathways such as *Piezo1*, thereby systematically reshaping the tumor microenvironment, remains largely unknown, and its underlying molecular mechanisms urgently need to be further investigated. In this study, transcriptome sequencing analysis was performed on SiHa cells cultured on gels with different matrix stiffness. The results showed that the transcriptional level of *IL-8* in the stiff gel group was significantly higher than that in the soft gel group (Figure 2D). We further detected the protein secretion level of *IL-8* in the cell culture supernatant by ELISA, and the results showed that the *IL-8* secretion of both cervical cancer cell lines in the stiff gel group was significantly higher than that in the soft gel group (Figure 2E), suggesting that increased matrix stiffness can significantly promote the transcription and expression of *IL-8* in cervical cancer cells. Through bioinformatics analysis using public databases, Kaplan–Meier survival analysis results indicated that cervical cancer patients with high expression of *Piezo1* and *IL-8* had significantly shorter overall survival than those with low expression (Figure 2F,G).

### 2.3. Increased Matrix Stiffness Promotes IL-8 Secretion in Cervical Cancer Cells by Activating the Piezo1/NF-κB Signaling Axis

**Figure 3:**
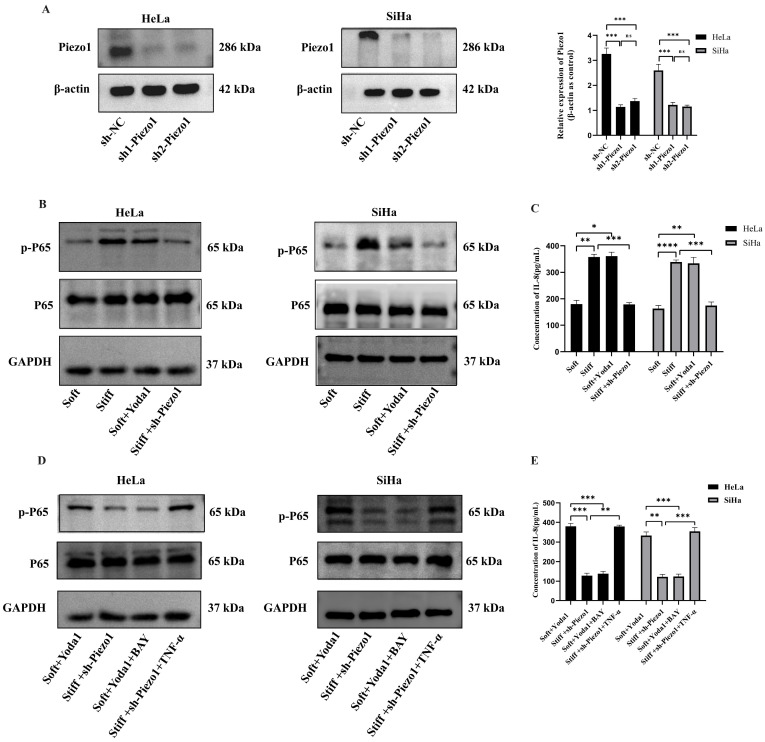
**The Piezo1/NF-κB signaling axis mediates matrix stiffness-regulated IL-8 secretion in cervical cancer cells.** (**A**). Western blot verification of the silencing efficiency of sh-Piezo1 on *Piezo1*; (**B**). Western blot and quantitative analysis of the effects of combined intervention of Piezo1 activation/knockdown and *NF-κB* modulation on *NF-κB* p65 phosphorylation and total protein expression under soft and stiff matrix conditions; (**C**). ELISA detection of the regulatory effect of the *Piezo1/NF-κB* signaling axis on *IL-8* secretion levels in cervical cancer cells under the same intervention conditions; (**D**). Western blot detection of the effects of Piezo1 agonist Yoda1 and *Piezo1* knockdown combined with *NF-κB* agonist/inhibitor treatment on phosphorylated p65 and total p65 protein expression under soft and stiff matrix conditions; (**E**). ELISA detection of the effects of Piezo1 agonist Yoda1 and Piezo1 knockdown combined with *NF-κB* agonist/inhibitor on *IL-8* secretion levels. All cell-based experiments were performed with 3 independent biological replicates, and ≥3 technical replicates for each test. * *p* < 0.05; ** *p* < 0.01; *** *p* < 0.001; **** *p* < 0.0001, ns: no significance.

Although we have confirmed that matrix stiffness can simultaneously upregulate the expression of *Piezo1* and *IL-8* in cervical cancer cells, the specific regulatory relationship among matrix stiffness, *Piezo1*, and *IL-8*, particularly whether Piezo1 directly participates in regulating *IL-8* expression and its underlying molecular mechanisms, remains to be further investigated. As a key sensor of mechanotransduction, *Piezo1* can initiate downstream signaling cascades upon activation; the *NF-κB* pathway, a classic regulatory pathway for inflammatory cytokine secretion, has been shown to be regulated by Piezo1, and its activation depends on the phosphorylation modification of *NF-κB* [19]. Activated *NF-κB* translocates to the nucleus and stimulates the expression of various pro-inflammatory cytokines, including *IL-8* [20]. Based on the above clues, this study proposes the following hypothesis: the *Piezo1/NF-κB* signaling axis may mediate the regulatory effect of matrix stiffness on *IL-8* secretion in cervical cancer cells. To verify this hypothesis, we used lentivirus-mediated shRNA technology to knock down *Piezo1* expression in HeLa and SiHa cells. Western blot results showed that compared with the empty vector control group (sh-NC), the Piezo1 protein expression levels in both cell lines in the sh-Piezo1 group were significantly decreased (Figure 3A). To further explore the regulatory mechanism of matrix stiffness on *Piezo1* and its downstream signaling pathways, we detected the expression levels and activation status of related proteins in both soft and stiff gel culture systems. Western blot analysis results showed that compared with the soft gel culture group, the phosphorylated *NF-κB* p65 levels in HeLa and SiHa cells cultured on stiff gels were significantly increased, suggesting that increased matrix stiffness can promote the activation of the *NF-κB* signaling pathway. To further verify whether the regulation of *IL-8* by matrix stiffness depends on *Piezo1*-mediated signal transduction, we performed bidirectional interventions on Piezo1 activity: *Piezo1* agonist Yoda1 was added to cervical cancer cells cultured on soft gels, while Piezo1 expression was knocked down by lentiviral transfection in cells cultured on stiff gels. The results showed that after Yoda1 treatment in the soft gel group, the originally low-level p-*NF-κB* p65 protein expression was significantly upregulated (Figure 3B), and the IL-8 content in the cell supernatant also increased accordingly (Figure 3C); conversely, after Piezo1 knockdown in the stiff gel group, the originally high-level p-*NF-κB* p65 protein expression was significantly downregulated (Figure 3B), and the *IL-8* secretion level also decreased correspondingly (Figure 3C). The above results indicate that Piezo1 plays a key mediating role in matrix stiffness-induced *NF-κB* pathway activation and IL-8 secretion.

To confirm the specific role of the *Piezo1/NF-κB* signaling axis in regulating *IL-8* secretion, this study further conducted rescue experiments. In the soft gel culture system, cervical cancer cells were treated with Yoda1 alone or in combination with the *NF-κB* inhibitor BAY; in the stiff gel system, *NF-κB* agonist TNF-α was administered on the basis of *Piezo1* knockdown. Western blot results showed that Yoda1 treatment could significantly upregulate the phosphorylated *NF-κB* p65 protein level, and this effect could be completely reversed by BAY; in contrast, in *Piezo1*-knocked down cells on stiff gels, *p-NF-κB* p65 expression was significantly decreased, and TNF-α stimulation could effectively restore its phosphorylation level (Figure 3D). ELISA results further indicated that activating *Piezo1* could significantly promote *IL-8* secretion by cervical cancer cells, while *Piezo1* knockdown significantly inhibited its secretion; the *NF-κB* inhibitor could completely eliminate the increase in *IL-8* release induced by Piezo1 activation, and the *NF-κB* agonist could effectively reverse the decrease in *IL-8* secretion caused by *Piezo1* knockdown (Figure 3E). Taken together, these results confirm that increased matrix stiffness precisely regulates the expression and secretion of *IL-8* in cervical cancer cells through the *Piezo1/NF-κB* signaling axis.

### 2.4. IL-8 Promotes Cervical Cancer Lymph Node Metastasis by Activating Neutrophil Extracellular Trap Formation

**Figure 4:**
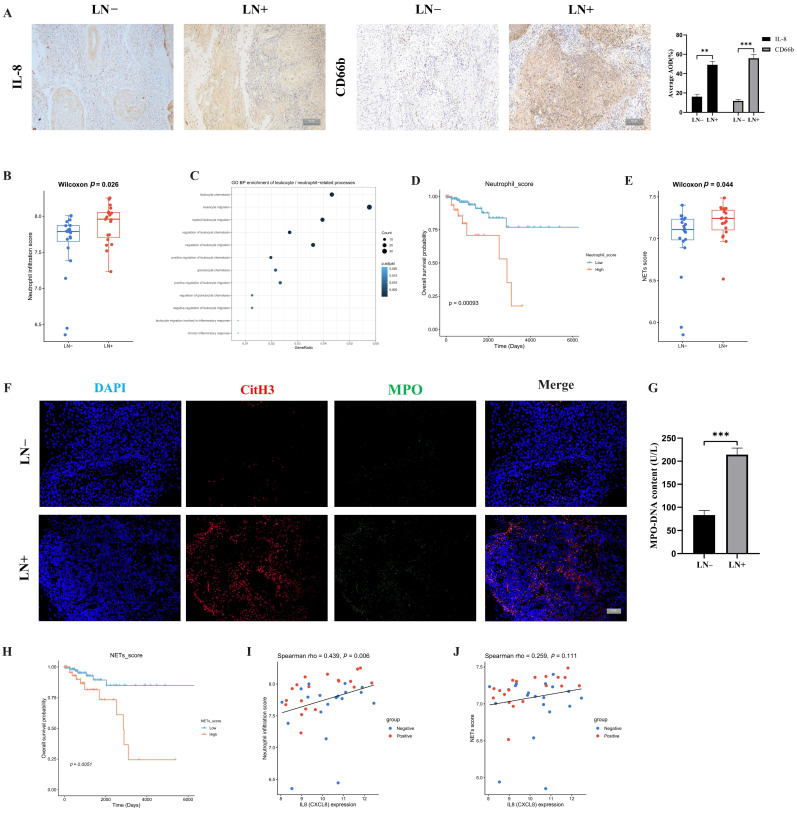
**Correlation Analysis of IL-8 Expression, Neutrophil Infiltration, and NETs Formation in Cervical Cancer Tissues with Lymph Node Metastasis.** (**A**). Immunohistochemistry assay detecting the expression levels of *IL-8* and neutrophil marker *CD66b* in cervical cancer tissues from the lymph node metastasis group and non-lymph node metastasis group (Scale bar: 50 μm); (**B**). In the GSE26511 dataset, bioinformatics analysis comparing neutrophil infiltration density in cervical cancer tissues between the lymph node metastasis group and non-lymph node metastasis group; (**C**). In the GSE26511 dataset, bioinformatics analysis of GO biological process enrichment analysis of differentially expressed genes in cervical cancer patients with or without pelvic lymph node metastasis; (**D**). In the TCGA database, bioinformatics analysis of the association between neutrophil infiltration density and overall survival (OS) of cervical cancer patients; (**E**). In the GSE26511 dataset, bioinformatics analysis comparing NETs-related scores in cervical cancer tissues between the lymph node metastasis group (LN+, red) and non-lymph node metastasis group (LN-, blue); (**F**). *MPO/CitH3* immunofluorescence colocalization assay detecting NETs formation in cervical cancer tissues from both groups (Scale bar: 50 μm); (**G**). ELISA assay detecting the levels of MPO-DNA complex, a specific marker of NETs, in the serum of patients from both groups; (**H**): In the TCGA database, bioinformatics analysis of the association between NETs_score and OS of cervical cancer patients; (**I**). Correlation analysis between *CXCL8* (*IL8*) expression and neutrophil infiltration score in the GSE26516 cohort; (**J**): Correlation analysis between *CXCL8* expression and NETs-related score. Tissue experiments: n = 10 per group (biological replicates), 5 random fields per sample; bioinformatic analyses from public databases without experimental repeats. ** *p* < 0.01; *** *p* < 0.001.

As an inflammatory factor in the tumor microenvironment, *IL-8* can specifically recruit neutrophils to infiltrate tumor tissues and activate neutrophils to release NETs [17]. Studies have confirmed that NETs can promote tumor lymph node metastasis by reshaping the tumor microenvironment and upregulating the expression of metastasis-related factors [21]. Therefore, we speculate that *IL-8* may mediate cervical cancer lymph node metastasis by regulating NETs formation. To verify this speculation, this study first conducted correlation analysis in clinical tissue samples. Immunohistochemistry results showed that the expression levels of *CD66b* (a neutrophil marker) and *IL-8* in cervical cancer tissues from the lymph node metastasis group were significantly higher than those from the non-lymph node metastasis group (Figure 4A); in the GSE26511 cohort, bioinformatics analysis results showed that patients with lymph node metastasis had higher neutrophil infiltration density in cervical cancer tissues (Figure 4B); enrichment analysis of differentially expressed genes in cervical cancer patients with or without lymph node metastasis showed that the differentially expressed genes were mainly enriched in leukocyte migration/activation processes related to leukocyte chemotaxis (Figure 4C); bioinformatics analysis results from TCGA data showed that high-density neutrophil infiltration was associated with poor prognosis in cervical cancer patients (Figure 4D); patients with lymph node metastasis had higher NETs-related scores in cervical cancer tissues (Figure 4E); *MPO/CitH3* immunofluorescence colocalization detection showed that the NETs-positive signal in tumor tissues from the lymph node metastasis group was significantly enhanced (Figure 4F); ELISA confirmed that the serum level of *MPO*-DNA, a specific marker of NETs, was also significantly increased in the lymph node metastasis group (Figure 4G); bioinformatics analysis results from TCGA data showed that high NETs-related scores were associated with poor prognosis in cervical cancer patients (Figure 4H); in the GSE26511 cohort, further correlation analysis showed that *CXCL8* (*IL-8*) expression was significantly positively correlated with neutrophil-related infiltration scores, suggesting that *IL-8* chemotactic signals may be related to enhanced neutrophil recruitment in cervical cancer (Figure 4I); meanwhile, there was only a weak positive correlation trend between *CXCL8* and NETs-related scores, which did not reach statistical significance (Figure 4J). These results indicate that *IL-8* expression is significantly increased in the cervical cancer lymph node metastasis group, and its high expression is closely related to increased neutrophil infiltration and enhanced NETs formation, which initially confirms the speculation that *IL-8* may mediate cervical cancer lymph node metastasis by regulating NETs formation.

**Figure 5:**
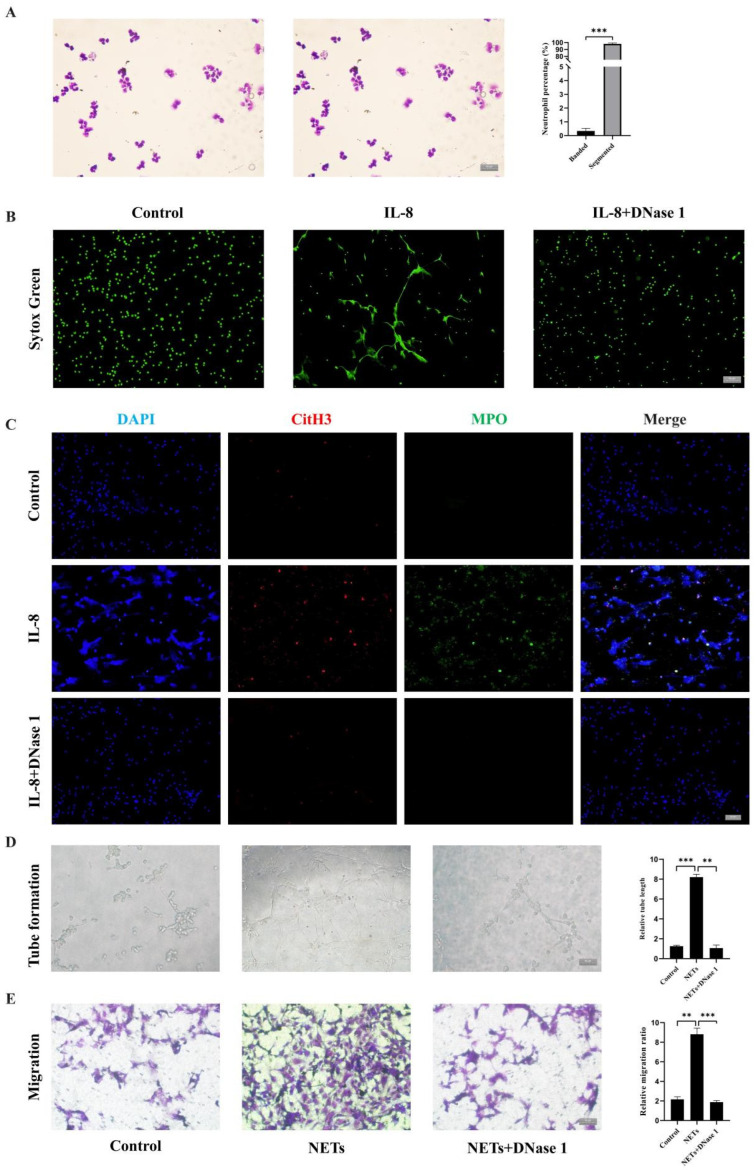
**IL-8 induces NETs formation and promotes tube formation and migration of HLECs.** (**A**). Giemsa staining of neutrophils isolated from human peripheral blood (left) and quantitative analysis of neutrophil purity (right); (**B**). NETs formation in neutrophils stimulated with recombinant IL-8 detected by Sytox Green staining; (**C**). Inductive effect of IL-8 on NETs formation in neutrophils verified by *MPO/CitH3* immunofluorescence co-localization (red: *CitH3*, green: *MPO*, blue: DAPI); (**D**). Capillary tube formation of HLECs on Matrigel after treatment with NETs-enriched supernatant of cervical cancer cells and quantitative analysis of total tubular network length (scale bar: 50 μm); (**E**). Migration ability of HLECs treated with the above supernatant examined by Transwell migration assay. All cell experiments: 3 independent biological replicates, ≥3 technical replicates each. ** *p* < 0.01; *** *p* < 0.001.

To determine whether *IL-8* promotes NET formation, human neutrophils were isolated from peripheral blood and purified. Giemsa staining confirmed that the purity of isolated neutrophils exceeded 95%, meeting the requirements for subsequent experiments (Figure 5A). Purified neutrophils were then stimulated with recombinant *IL-8*. Sytox Green staining showed that the proportion of cells with reticular fluorescent structures was significantly higher in the *IL-8* stimulated group than in the control group (Figure 5B). Immunofluorescence co-localization of *MPO* and *CitH3* further verified that *IL*-8 effectively induced neutrophils to produce typical NET structures, suggesting that *IL-8* is a key inflammatory cytokine regulating NET formation in the tumor microenvironment (Figure 5C). To clarify the effect of NETs on cervical cancer lymph node metastasis, NETs induced by *IL-8* were collected, and NETs-containing supernatants were applied to human lymphatic endothelial cells (HLECs). Tube formation assay revealed that the total length of capillary-like networks formed by HLECs on Matrigel was significantly longer in the NETs-treated group than in the control group (Figure 5D). Transwell migration assay demonstrated that the migration ability of HLECs was markedly enhanced after NETs treatment (Figure 4E). Collectively, these results indicate that NETs potentiate tube formation and migration capabilities of HLECs.

### 2.5. Reducing Matrix Stiffness Inhibits Cervical Cancer Lymph Node Metastasis In Vivo

**Figure 6:**
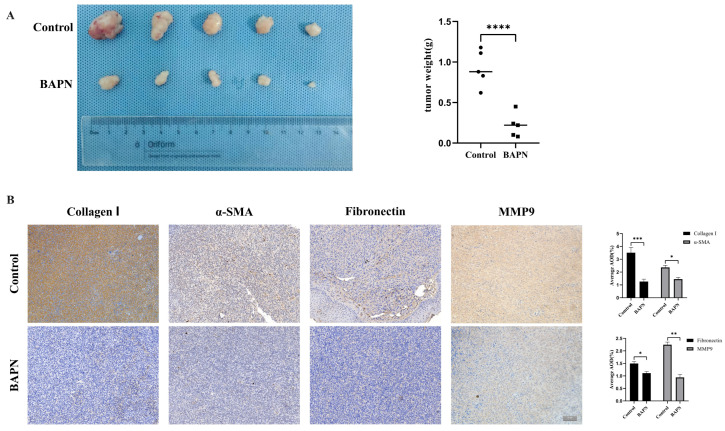
**Reducing matrix stiffness inhibits cervical cancer tumor growth in vivo.** (**A**). Mouse footpad lymph node metastasis model: control group (n = 5) received PBS, experimental group (n = 5) received BAPN (50 mg/kg), and tumor size and weight were compared between the two groups; (**B**). Expression levels and quantitative analysis of *Collagen I*, *α-SMA*, *Fibronectin* and *MMP9* in mouse tumor tissues detected by immunohistochemistry (scale bar: 50 μm). n = 5 mice per group as biological replicates; 5 random fields per sample for technical quantification. * *p* < 0.05; ** *p* < 0.01; *** *p* < 0.001; **** *p* < 0.0001.

In this study, a cervical cancer lymph node metastasis model was established by inoculating TC-1 cells into the footpads of female C57BL/6 mice. Tumor-bearing mice were randomly divided into two groups: the experimental group received intraperitoneal injection of the lysyl oxidase irreversible inhibitor β-aminopropionitrile (BAPN, 50 mg/kg) to disrupt collagen cross-linking and reduce matrix stiffness, while the control group received an equal volume of PBS. Mice were sacrificed after 4 weeks of administration, and tumor tissues were collected for subsequent assays. Compared with the control group, the volume and weight of orthotopic footpad tumors were significantly decreased in the BAPN-treated group (Figure 6A). Immunohistochemical results showed that the expression levels of matrix stiffness-related markers, including *Collagen I*, *α-SMA*, *Fibronectin* and *MMP9*, were markedly downregulated in tumor tissues after BAPN intervention (Figure 6B).

**Figure 7:**
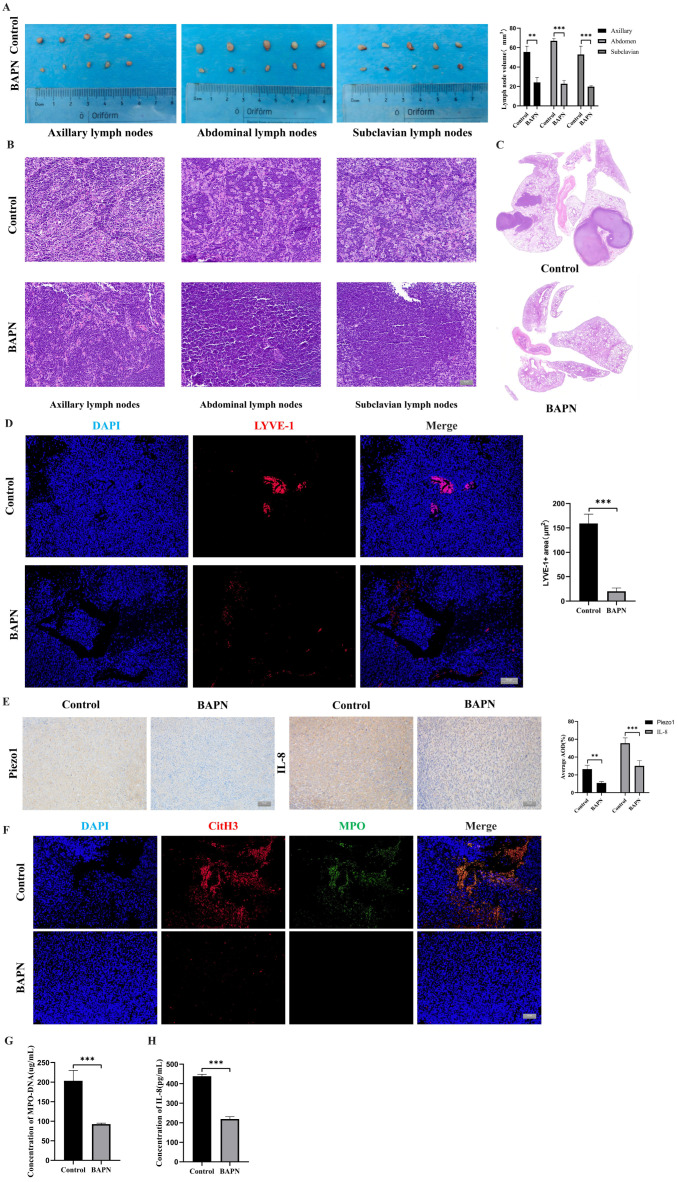
**Reducing matrix stiffness inhibits lymphangiogenesis, lymph node metastasis and lung metastasis of cervical cancer in vivo.** (**A**). Comparison of regional lymph node volumes between the two groups of mice; (**B**). Representative HE staining images of lymph nodes in the lymph node metastasis model; (**C**). Observation of metastatic foci in lung tissues by HE staining; (**D**). Detection of lymphatic vessel density in tumor tissues of the two groups of mice by *LYVE-1* immunofluorescence staining(Red: *LYVE-1*, Blue: DAPI); (**E**). Detection of *Piezo1* and *IL-8* expression levels in tumor tissues of the two groups of mice by immunohistochemistry; (**F**). Detection of NETs expression in tumor tissues of the two groups of mice by tissue immunofluorescence staining (Red: *CitH3*, Green: *MPO*, Blue: DAPI); (**G**). Serum ELISA assay and quantitative analysis of MPO-DNA complex, a specific marker of NETs, in the serum of the two groups of mice; (**H**). Serum ELISA assay and quantitative analysis of *IL-8* expression levels in the serum of the two groups of mice. n = 5 mice per group as biological replicates; 5 random fields per sample for technical quantification. ** *p* < 0.01; *** *p* < 0.001.

Further studies revealed that reducing matrix stiffness not only inhibited the growth of orthotopic tumors but also had a significant impact on the metastatic potential of cervical cancer. The volumes of popliteal, abdominal, and supraclavicular lymph nodes in mice from the BAPN experimental group were significantly reduced (Figure 7A). Further confirmation by HE staining showed that the lymph node metastasis rate was significantly decreased in the BAPN group (Figure 7B), and HE staining of lung tissues confirmed that the number of intrapulmonary metastatic foci was significantly reduced in the BAPN experimental group (Figure 7C). Immunofluorescence results showed that the lymphatic vessel density in tumor tissues from the BAPN experimental group was significantly lower than that in the control group (Figure 7D). These results indicated that reducing matrix stiffness could effectively inhibit lymph node metastasis of cervical cancer. We detected the expression of *Piezo1*, *IL-8*, and the formation of NETs in tumor tissues. Immunohistochemistry results showed that the expression levels of *Piezo1* and *IL-8* in cervical cancer tissues from the BAPN group were significantly lower than those in the control group (Figure 7E); immunofluorescence results showed that the level of NETs formation in tumor tissues from the BAPN experimental group was significantly reduced (Figure 7F); the levels of MPO-DNA (a specific marker of NETs) and *IL-8* in the serum of the BAPN experimental group were also significantly decreased (Figure 7G,H). These results confirmed that reducing matrix stiffness could effectively inhibit the formation and release of NETs in the tumor microenvironment, thereby inhibiting lymph node metastasis of cervical cancer.

## 3. Discussion

Cervical cancer, as the most common gynecological malignancy, its recurrence and metastasis remain core challenges in clinical treatment. According to the 2020 global cancer statistics, there were 604,000 new cases and 342,000 deaths worldwide, with 85% occurring in developing countries [22]. After systemic treatment for advanced cervical cancer, 28–64% of patients still experience recurrence, and once recurrence or metastasis occurs, the survival rate of cervical cancer patients decreases significantly [23]. Therefore, exploring the molecular mechanisms of recurrence and metastasis in advanced cervical cancer is of great significance for improving the therapeutic effect and prognosis of patients with advanced cervical cancer.

Clinical observations have found that tumor tissues generally harden and matrix stiffness increases significantly during the recurrence and metastasis of cervical cancer. In this study, quantitative measurement by atomic force microscopy revealed that the matrix stiffness of lymph node metastatic cervical cancer tissues was significantly higher than that of non-metastatic tissues. This finding indicates a significant positive correlation between elevated matrix stiffness and the metastatic potential of cervical cancer. However, it is important to distinguish that analyses of human samples only reveal such correlations, while the establishment of a causal relationship relies on subsequent validation using experimental models. As a core physical characteristic of the tumor microenvironment, matrix stiffness (MS) has been confirmed to regulate malignant phenotypes such as chemotherapy resistance, invasion and metastasis of tumor cells, and plays a key role in tumor progression [24]. However, current research on the tumor microenvironment mostly focuses on changes in extracellular matrix components and biochemical molecules, and there is still a lack of systematic research on the function and mechanism of the mechanical microenvironment and tumor cell mechanotransduction in tumor progression. In particular, tumor biomechanics research represented by MS has become a new hotspot in tumor microenvironment research [25]. Only a few studies have shown that increased MS can upregulate the Young’s modulus and cytoskeletal mechanical properties of cervical cancer cells [26], but the specific regulatory role and molecular mechanism of MS in cervical cancer lymph node metastasis have not been clarified. In vivo experiments in this study confirmed that the matrix softener BAPN can effectively inhibit cervical cancer cell growth, reduce tumor volume, and decrease lymph node metastasis. This provides direct experimental evidence supporting the causal inference that “elevated MS is a key driving factor for cervical cancer lymph node metastasis,” laying a foundation for subsequent mechanism exploration.

To screen for downstream key molecules regulated by MS in cervical cancer metastasis, this study first performed transcriptome sequencing on cervical cancer cells cultured on polyacrylamide gels of varying matrix stiffness. The results found that stiff matrices can significantly upregulate the synthesis and secretion of the pro-inflammatory chemokine *IL-8*. The mechanosensitive ion channel *Piezo1* is a core protein that senses extracellular mechanical stimuli, can specifically recognize changes in matrix stiffness, convert them into intracellular chemical signals, and participate in regulating processes such as tumor metabolic reprogramming, inflammatory response, and tumor microenvironment remodeling [27]. Existing studies have shown that elevated MS can trigger downstream signals by activating Piezo1 to regulate tumor progression [28]. This study also confirmed that high stiffness matrices can activate the expression of Piezo1 in cervical cancer cells. Clinical sample validation showed that the expression of *IL-8* and Piezo1 in lymph node metastatic cervical cancer tissues was significantly higher than that in non-lymph node metastatic cervical cancer tissues, consistent with the trend of elevated MS. This finding for the first time establishes an association between matrix stiffness and *Piezo1/IL-8* in clinical samples and preliminarily validates their regulatory relationship using cellular models, providing a key entry point for elucidating the pro-metastatic molecular mechanism of MS.

To explore the potential association between Piezo1 and the upregulation of *IL-8* expression, this study conducted functional verification. The results showed that high stiffness matrices can activate the expression of Piezo1 in cervical cancer cells, while knocking down *Piezo1* can significantly inhibit the *IL-8* elevation induced by stiff matrices, suggesting that Piezo1 may be a key molecule for MS to regulate *IL-8*.

To further clarify the molecular mechanism by which Piezo1 regulates *IL-8*, we focused on *NF-κB*, a core transcription factor of the inflammatory pathway. *NF-κB* plays a key role in the construction of the inflammatory microenvironment and disease progression of cervical cancer [29]. In the resting state, *NF-κB* binds to inhibitory proteins and is localized in the cytoplasm; after activation, its phosphorylation level increases significantly, which in turn binds to the promoter/enhancer regions of inflammatory factor genes such as *IL-8* to regulate target gene transcription [30]. Verification in this study found that after activation of *Piezo1* by high stiffness matrices, the activation level of *NF-κB* (manifested as increased phosphorylation status) was significantly enhanced; conversely, *Piezo1* knockdown significantly inhibited the activation of *NF-κB* and reduced the transcription and secretion of *IL-8*. This result clearly links the matrix stiffness/*Piezo1/NF-κB/IL-8* regulatory axis, clarifying the molecular mechanism by which matrix stiffness regulates *IL-8* expression.

Interleukin-8 is a classic pro-inflammatory chemokine. Studies have found that *IL-8* can enhance inflammatory responses in the tumor microenvironment, promote pre-metastatic niche formation, and inhibit anti-tumor immunity by recruiting neutrophils and inducing their release of neutrophil extracellular traps (NETs), thereby accelerating tumor progression and metastasis [31]. The discovery of NETs provides a new perspective for oncology research, prompting people to re-examine the role of neutrophils in the tumor microenvironment, no longer simply regarding them as transient effector cells. Neutrophil extracellular traps are reticular structures released by neutrophils, mainly composed of DNA, histones, myeloperoxidase, and neutrophil elastase [32]. In recent years, more and more studies have shown that NETs are closely related to the malignant progression of tumors [33]. Specifically, NETs can not only physically trap circulating tumor cells through their reticular structures but also form a protective barrier to protect tumor cells from attack by cytotoxic T cells and natural killer cells [21]. For example, *S100A7* promotes cervical cancer lymph node metastasis through a dual mechanism of synergistically regulating neutrophil chemotactic recruitment and NETs formation [16]. Although existing studies have revealed the role of NETs in tumor metastasis, the specific mechanisms by which NETs regulate cervical cancer proliferation and distant metastasis, particularly through promoting lymphangiogenesis, remain to be further elucidated. This study demonstrated that the level of NETs in metastatic cervical cancer tissues was significantly higher than that in non-metastatic cervical cancer tissues; in vitro experiments proved that *IL-8* can effectively promote neutrophils to form NETs, and NETs can enhance the tube formation and migration abilities of lymphatic endothelial cells, ultimately promoting lymphangiogenesis and metastasis of cervical cancer.

In summary, from the perspective of tumor biomechanical signal transduction, this study proposes the following core hypothesis: in cervical cancer, increased matrix stiffness acts as a key physical driving force that can activate the mechanotransduction molecule Piezo1, promote the activation of the *Piezo1/NF-κB* pathway, and induce *IL-8* release; *IL-8* further recruits and activates neutrophils, induces the formation of neutrophil extracellular traps, and ultimately promotes cervical cancer lymph node metastasis. This study reveals the key role of the mechanical microenvironment as a mechanical driving factor in cervical cancer metastasis, providing new potential targets for the treatment of advanced cervical cancer.

However, this study still has certain limitations. Firstly, while the polyacrylamide gel system used in this study is a classic tool for investigating the effects of matrix stiffness, its representation of the tumor microenvironment is overly simplistic. The real tumor microenvironment is a highly complex system comprising diverse extracellular matrix components, cellular heterogeneity, and dynamically changing biomechanical properties, complexities that are not fully recapitulated in gel models. Secondly, although we confirmed that Piezo1 activates the downstream *NF-κB* pathway, the specific molecular transduction process from mechanical signal perception by *Piezo1* to the activation of the *NF-κB* complex requires further exploration. Additionally, the downstream target molecules regulated by NETs formation in cervical cancer metastasis have not been clarified, which need to be further investigated to improve the mechanism chain. Future research could employ more complex 3D tumor models or organoid models, integrating techniques such as single-cell sequencing and protein interaction analysis to more deeply unravel this complex regulatory network. Concurrently, the possibility that matrix stiffness may regulate tumor metastasis through *Piezo1*-independent signaling pathways should also be considered and warrants further investigation.

## 4. Materials and Methods

### 4.1. Patients and Tissue Samples

Twenty patients with cervical cancer were enrolled at Renmin Hospital of Wuhan University between January 2025 and July 2025. Samples were classified into lymph node metastatic and non-metastatic groups. The study was approved by the Ethics Committee of Renmin Hospital of Wuhan University (No. WDRY2024-K178) and performed in accordance with the Declaration of Helsinki. Written informed consent was obtained from all participants.

### 4.2. Cell Lines and Isolation of Primary Neutrophils

Human cervical cancer cell lines HeLa, SiHa, and TC-1 were obtained from the Cell Bank of Wuhan University. Human neutrophils were isolated from peripheral blood of healthy volunteers using a commercial isolation kit (Solarbio Science & Technology Co., Ltd., Beijing, China).

### 4.3. Animals and In Vivo Tumor Model

A total of 10 female SPF-grade C57BL/6 mice (6–8 weeks old, weight 18–22 g) were purchased from Guangdong Yaokang Biotechnology Co., Ltd. (Guangzhou, Guangdong, China). All animal experimental protocols were approved by the Animal Ethics Committee of Renmin Hospital of Wuhan University. Mice were adaptively fed for 1 week under SPF conditions before the experiment. TC-1 cells were cultured to logarithmic phase, harvested, and resuspended in pre-cooled PBS to a final concentration of 1 × 10^7^ cells/mL. A 50 μL aliquot of the cell suspension was injected into the right hind footpad of each mouse to establish a cervical cancer lymph node metastasis model. After inoculation, mice were randomly assigned into two groups (n = 5 per group) using a random number table: the PBS control group and the BAPN treatment group. To ensure blinding, the investigator responsible for drug administration and data collection was unaware of the group allocation. The same investigator also performed tumor measurement and outcome assessment under blinded conditions. Mice were randomly divided into two groups (n = 5 per group): the PBS control group and the BAPN treatment group. Mice in the BAPN group were intraperitoneally injected with 50 mg/kg BAPN (MedChemExpress, Monmouth Junction, NJ, USA) every 2 days, while the control group received an equal volume of PBS. During the 4-week treatment period, mouse body weight, footpad tumor growth, and general condition were monitored every 2 days, and tumor length and width were measured to evaluate tumor volume. At the end of the experiment, mice were sacrificed, and orbital blood, footpad tumor tissues, popliteal lymph nodes, abdominal lymph nodes, subclavian lymph nodes, and lung tissues were collected. Tumor tissues were split into two parts: one part was fixed in 4% paraformaldehyde for paraffin embedding and histological staining, and the other part was rapidly frozen in liquid nitrogen for subsequent molecular experiments.

### 4.4. Atomic Force Microscopy

Fresh cervical cancer tissue samples were cut into 30 μm-thick continuous sections, which were then immobilized on sterile cell culture dishes coated with a thin layer (<5 μm) of epoxy glue to ensure tight adhesion without wrinkles or damage. Pre-cooled lactated Ringer’s solution containing 1% protease inhibitor was added to the dishes to keep the sections moist. Young’s modulus was measured using a Bruker atomic force microscope (Bruker Corporation, Billerica, MA, USA) in fluid contact mode under near-physiological conditions. A DNP-10 triangular silicon cantilever (Bruker Corporation, Billerica, MA, USA) (k = 0.06 N/m, f_0_ = 18–65 kHz, cantilever length 8.0 μm, tip radius ≈ 20 nm, θ = 17.5° ± 2.5°) was used. The scanning parameters were set as follows: maximum normal force 1–1.4 nN, *Z*-axis cycle frequency 0.5 Hz. Before measurement, the cantilever spring constant and detector sensitivity were calibrated by the thermal noise method to ensure accurate Young’s modulus inversion. For each section, force spectroscopy data were recorded at ≥3 random sites, with 5–8 sets of data collected per site.

### 4.5. Histological Staining

Paraffin-embedded tissues were subjected to hematoxylin and eosin (HE), Masson’s trichrome, and Sirius Red staining. Immunohistochemistry (IHC) was performed to detect *Fibronectin*, *α-SMA*, *Collagen I*, *MMP9* and *LYVE-1*. Immunofluorescence staining was used to visualize NETs (MPO/CitH3) and lymphatic vessels.

### 4.6. Cell Culture and Transfection

HeLa and SiHa cells were cultured in DMEM/high glucose medium supplemented with 10% FBS and 1% penicillin-streptomycin, and passaged when reaching 80–90% confluence. For Piezo1 knockdown, cells were seeded in 6-well plates and infected with lentiviruses expressing *Piezo1* short hairpin RNA (shRNA, target sequence: 5′-GCGTCATCATCGTGTGTAAGA-3′ and 5′-GCTACGAGAACAAGCCCTACT-3′) or non-targeting control shRNA (5′-TTCTCCGAACGTGTCACGT-3′) when cell confluence reached 20–30%. After 72 h of infection, positive cells were selected with 2 μg/mL puromycin (HeLa) or 0.5 μg/mL puromycin (SiHa) for 48 h. The knockdown efficiency of Piezo1 was verified by Western blot. For drug treatment, cells were treated with Yoda1 (50 μM), BAY (1 μM), or TNF-α (10 ng/mL) 8–12 h after seeding, and cultured for the indicated time before subsequent experiments.

### 4.7. Polyacrylamide Gel Substrates

Polyacrylamide gels with two different Young’s moduli were prepared: soft gels (~19 kPa, 8% acrylamide and 0.26% bis-acrylamide) and stiff gels (~90 kPa, 12% acrylamide and 0.28% bis-acrylamide). Briefly, acrylamide and bis-acrylamide stock solutions were mixed according to the desired concentrations, and 1/100 volume of ammonium persulfate (AP) and 1/1000 volume of TEMED were added to initiate polymerization. The gel mixture (400 μL per well) was quickly added to silanized slides and covered with silanized coverslips. After polymerization for 5 min, coverslips were gently removed, and gels were washed with PBS containing 1% penicillin-streptomycin. Gels were activated with Sulfo-SANPAH under UV irradiation for 10 min, then coated with 0.2 mg/mL type I collagen at 4 °C overnight. Before cell seeding, gels were washed with PBS and equilibrated in complete medium at 37 °C for ≥30 min. HeLa and SiHa cells were seeded on the gels and cultured for 72 h for subsequent experiments.

### 4.8. RNA Sequencing

HeLa and SiHa cells were cultured on soft or stiff polyacrylamide gels for 72 h, and total RNA was extracted using Trizol reagen (Servicebio, Wuhan, Hubei, China). RNA quality and integrity were verified by agarose gel electrophoresis and a NanoDrop spectrophotomete (Thermo Fisher Scientific, Waltham, MA, USA). RNA-seq was performed on an Illumina Novaseq sequencing platform by a commercial service. Raw sequencing data were standardized, and differentially expressed genes (DEGs) were identified using DESeq2 software (version 1.34.0), with |log2(fold change)| > 1 and *p* < 0.05 as the screening criteria.

### 4.9. Bioinformatics Analysis

Data from GEO dataset GSE26511 (39 cervical cancer samples) were retrieved and processed, including gene expression and clinical data. Samples were grouped by lymph node metastasis status (Negative, n = 20; Positive, n = 19). DEGs were screened using DESeq2 (*p* < 0.05, |logFC| ≥ 1), followed by GO enrichment analysis with clusterProfiler. Neutrophil and NETs scores were calculated using established gene signatures. TCGA data were used for OS analysis of *IL8*, Neutrophil_score, and NETs_score. Patients were stratified using the optimal cutpoint determined by the survminer package (for *CEACAM8*, expression >0 vs. =0). Univariate Cox regression was performed. Due to the limited number of death events, multivariate Cox regression adjusting for covariates was not performed to avoid model overfitting. Spearman correlation was used to analyze the relationship between *CXCL8* expression and scores in GSE26511.

### 4.10. Western Blotting

Total protein was extracted using RIPA lysis buffer. Protein concentrations were determined by BCA assay. Samples were separated by SDS-PAGE, transferred to PVDF membranes, and probed with primary antibodies against *Piezo1*, *NF-κB p65*, *p-p65*, and *IL-8*, followed by HRP-conjugated secondary antibodies. Signals were visualized using ECL reagen (Abbkine Scientific Co., Ltd., Wuhan, Hubei, China).

### 4.11. NET Induction and Identification

Isolated neutrophils were seeded in 12-well plates at a density of 2 × 10^5^ cells per well and stimulated with 50 ng/mL *IL-8* (MedChemExpress, USA) at 37 °C with 5% CO_2_ for 4 h to induce NET formation. NET formation was verified by Sytox Green staining (KeyGen BioTECH, Nanjing, Jiangsu, China) and immunofluorescence double staining for *MPO* and *CitH3*. The criteria for NET positivity were defined as the presence of extracellular, web-like fibrous structures formed by the co-localization of DNA (stained by DAPI) with *MPO* or *CitH3* signals under immunofluorescence microscopy. A positive Sytox Green staining was indicated by the appearance of web-like green fluorescent signals outside the cells. For isolation of cell-free NETs, culture supernatants were collected, centrifuged at 450× *g* for 10 min at 4 °C to remove neutrophils, and the supernatant was further centrifuged at 18,000× *g* for 10 min to precipitate NETs. The pellet was resuspended in pre-cooled PBS, and DNA concentration was measured using a spectrophotometer to ensure it ranged from 140 to 180 ng/μL.

### 4.12. Tube Formation and Transwell Assays

For the tube formation assay, 96-well plates and pipette tips were pre-cooled, and Matrigel was melted at 4 °C overnight. Fifty microliters of Matrigel was added to each well and allowed to solidify at 37 °C for 30 min. Human lymphatic endothelial cells (HLECs) were digested with trypsin, resuspended in complete medium, and seeded on Matrigel at a density of 1 × 10^4^ cells per well. Cells were incubated with NET-conditioned medium for 6 h, and tube formation was observed under an inverted microscope. Three random fields were selected per well, and the total length of tube networks was measured for statistical analysis. For the Transwell assay, HeLa and SiHa cells were resuspended in serum-free DMEM at a density of 2 × 10^5^ cells per well and seeded in the upper chamber of Transwell inserts (Corning Incorporated, Corning, NY, USA). The lower chamber was filled with 800 μL of DMEM containing 20% FBS. NETs or DNase I was added to the upper chamber as indicated, and cells were cultured for 48 h. Non-migrated cells in the upper chamber were wiped off, and migrated cells were fixed with 4% polyacrylamide, stained with 0.1% crystal violet, and observed under an inverted microscope at 200× magnification.

### 4.13. ELISA

Concentrations of human and mouse *IL-8* and MPO-DNA complexes in serum or culture supernatants were measured using commercial ELISA kits according to the manufacturer’s instructions.

### 4.14. Statistical Analysis

All experiments were performed independently at least three times, and data are presented as the mean ± standard deviation (SD). Statistical analyses were performed using SPSS 21.0 software. Comparisons between two groups were analyzed using unpaired Student’s *t*-test, and comparisons among multiple groups were analyzed using one-way analysis of variance (ANOVA) followed by Tukey’s post hoc test. Image processing was performed using Photoshop 2019 and GraphPad Prism 9.0. A *p* value < 0.05 was considered statistically significant.

## Data Availability

The original contributions presented in this study are included in the article. Further inquiries can be directed to the corresponding author.
